# Pineal Gland Agenesis: Review and Case Illustration

**DOI:** 10.7759/cureus.1314

**Published:** 2017-06-05

**Authors:** Marcus A Cox, Michele Davis, Vlad Voin, Mohammadali Shoja, Rod J Oskouian, Marios Loukas, R. Shane Tubbs

**Affiliations:** 1 Medical Education, Saint Michael's Medical Center; 2 Department of Anatomical Sciences, St. George's University School of Medicine, Grenada, West Indies; 3 Research Fellow, Seattle Science Foundation; 4 Surgery, University of Texas Medical Branch at Galveston; 5 Swedish Neuroscience Institute; 6 Department of Anatomical Sciences, St. George's University School of Medicine, Grenada, West Indies; 7 Neurosurgery, Seattle Science Foundation

**Keywords:** pineal gland, embryology, brain, diencephalon, aplasia, variation

## Abstract

Agenesis of the pineal gland has rarely been reported in the medical literature. Herein, we report a cadaveric specimen found to have agenesis of the pineal gland. The remaining gross examination of the brain was normal. A review of the literature was performed on this unusual finding.

## Introduction

The pineal gland is a neuroendocrine organ which develops from the diencephalon. Although the primary function is the secretion of melatonin, the role in human growth and development has not been clearly defined. Although exceptionally rare, the absence of the pineal gland has been recorded as an incidental finding on magnetic resonance imaging (MRI). It has been linked to the PAX6 gene mutation, which is a transcription factor [1-2]. In this article, we will review rare cases of pineal agenesis and their associated syndromes, with special attention to ocular and brain abnormalities in patients with PAX6 mutations. A cadaveric specimen with pineal gland agenesis will be presented as an illustration.

## Case presentation

**Figure 1:**
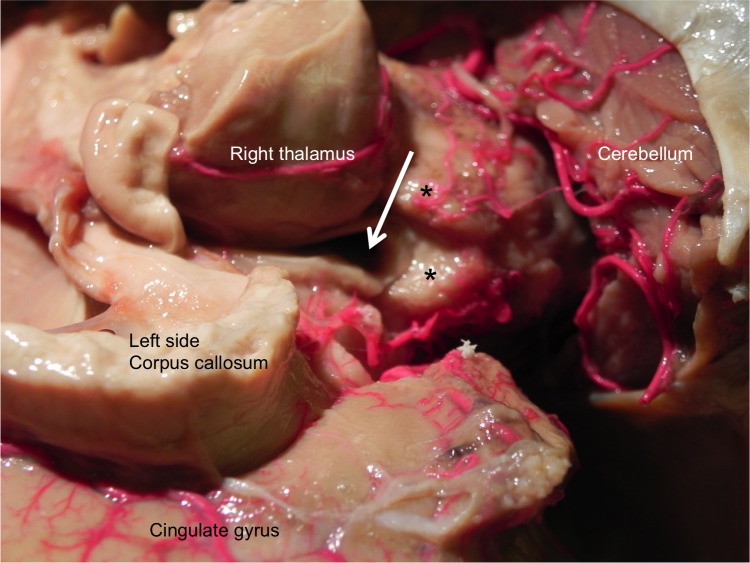
Absent Pineal Gland Left posterolateral view of the brain of the case illustration herein. Notice the absence of the pineal gland.

During routine dissection of a Caucasian male cadaver (79 years old at death), the absence of the pineal gland was noted (Figure 1). The patient had a history of hypertension, osteoarthritis, and glaucoma. He expired due to a myocardial infarction. No other medical history was reported including any syndromes or previous intracranial surgery. The patient had a left inguinal region incision and had undergone a left knee replacement.

During the dissection of the cranium and its contents, no other intracranial anatomical variations were noted. Specifically, the other diencephalic derived brain structures (e.g., thalamus, optic nerves, hypothalamus, posterior pituitary gland) were all within normal limits. No pathology of the brain was noted such as hydrocephalus or ectopic tissues.

## Discussion

The pineal gland develops as an evagination of the neuroepithelium from the dorsal diencephalon, above the future third ventricle, and has been attributed as the main producer of melatonin. The shape of the pineal gland in mammals is variable but usually presents as a lobular structure. From early human life the pineal gland begins to calcify and calcification increases with age, which may correlate with the age-related decline in melatonin production. Decreases in the night time levels of this hormone may be responsible for fragmented sleep-wake patterns [3].

Pineal gland agenesis has been observed in murine and humans with mutations in the paired box gene 6 (PAX6), where the pineal gland along with other brain and eye abnormalities has been observed [1, 4-5]. PAX6 is expressed in the telencephalon, diencephalon, caudal part of the rhombencephalon, myelencephalon, the spinal cord and pancreas, explaining the various phenotypes observed in individuals with mutations in this gene [1, 4-5]. PAX6 has been shown to play an important role as a regulatory gene in cortical developmental processes including cellular proliferation, neuronal migration, and axonal guidance [1, 6-7]. The mechanism by which PAX6 expression influences structural formation is unknown, but one explanation is that PAX6 regulates expression of a gene directing synthesis of R-cadherin and other cell surface recognition molecules [8]. Mapping results of the PAX6 gene by Thakurela, et al., demonstrated a dual function of PAX6 upon neuronal commitment where it mediates the activation of neuronal genes while concurrently suppressing the mesodermal and endodermal genes to ensure the unidirectionality toward neuronal differentiation [9]. They also concluded that PAX6 induces critical signaling pathways that further work together in guiding critical neurogenic events such as neurogenesis, neuronal differentiation, and lens development [9].

In mice, PAX6 is critical for survival. PAX6 null mice die immediately after birth [5-6]. Heterozygote mice have a subtle form of cortical and eye abnormalities. There have only been a few cases reported in humans with mutations in both PAX6 alleles. In these cases, patients had severe brain and eye abnormalities reported [5, 7]. Human heterozygotes have a variety of anomalies [1-2, 6-7]. Studies have been performed using imaging techniques in patients with PAX6 mutation to identify the extent of these abnormalities.

High-resolution MRIs performed on 24 individuals with ocular abnormalities and positive PAX6 mutations showed 13 individuals had absent pineal glands (54%), four had structurally normal pineal glands (17%) and the remaining seven had pineal glands which were difficult to visualize and were deemed “hypoplastic” (29%) [1]. Although this study shows a link between PAX6 mutations, aniridia, and pineal agenesis, it is limited by the small sample size and the possibility that pineal tissue may be demonstrable histopathologically while not being visualized on MRIs. Abouzeid, et al. showed that out of 10 patients from three different families in Egypt who had PAX6 mutations, all 10 had bilateral aniridia while three had absent pineal glands (30%) [4]. This was much less than the 54% in the study of Mitchell, et al. who had both pineal agenesis and ocular abnormalities [1]. All patients in the study published by Abouzeid, et al. had a full ophthalmic and neurological examinations performed at the time and showed no major neurodevelopmental delays [4]. Although the sample size remains small, the current evidence points to a distinct link between ocular abnormalities and pineal agenesis with PAX6 mutations.

Yogarajah, et al. investigated MRI parameters in 19 adults with known PAX6 mutations to understand the importance that the PAX6 gene plays in the maintenance of brain integrity. They found in people with heterozygous mutation in PAX6, there was exaggerated cortical thinning with age, a reduction in cortical thickness that correlated with a decline in working memory, and abnormalities of cortical patterning. They concluded that these findings could play an important role in understanding neurodegenerative disorders and modulation of PAX6 may offer new therapeutic strategies in fighting these diseases [5].

Ellison-Wright, et al. demonstrated significant reductions of white matter in the anterior and posterior corpus callosum, local excesses of grey matter in regions including the hippocampus, ventral striatum, insula, and cerebellum in humans with heterozygous PAX6 mutation [8]. Free, et al. established variations in cortical structures in patients with a PAX6 abnormality using quantitative MRI analysis [7]. Sisodiya, et al. observed that patients with the PAX6 mutation had cerebral malformation and olfactory dysfunctions [10]. These findings show there is a link between people with PAX6 mutations and abnormal brain development.

Being a transcription factor, PAX6 interacts with several brain developmental genes and other transcription factors. With the development of high-resolution MRIs, the extent of abnormalities in these patients is being examined more closely.

## Conclusions

The extent to which pineal agenesis affects human growth and development has not been explored in the articles reviewed. Extensive neurological, endocrine, and sleep studies on these patients might help provide further insight into the function of this gland in normal human growth and development. Also, the PAX6 gene plays an important role in brain development and formation. While the correlation between PAX6 gene mutation and pineal agenesis is not definite, there seems to be a link between patients with pineal agenesis and mutations in the PAX6 gene. Further study of this gene could lead to novel treatments and our understanding of neurodegenerative diseases.
